# Supplementary material for: High-throughput proteome integral solubility alteration assay for low cell input using One-Tip
Source: Commun Chem. 2025 Sep 26;8:282. doi: 10.1038/s42004-025-01670-4 (PMC12474936; doi:10.1038/s42004-025-01670-4)
Supplement: Supplementary file 2 — Supplementary information [file 42004_2025_1670_MOESM2_ESM.pdf]

Supplementary figures

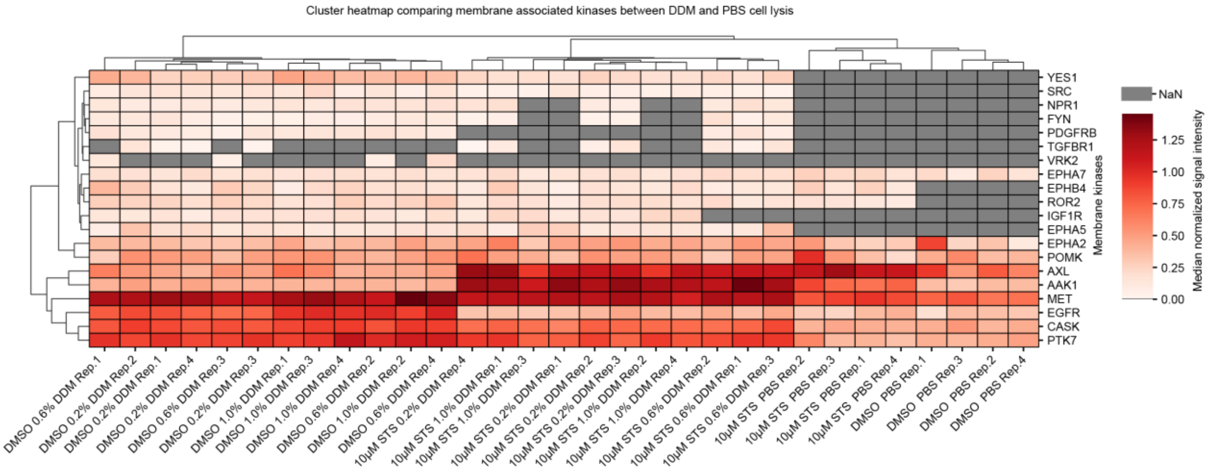

Supplementary Figure 1. Cluster heatmap for all DDM and PBS cell lysis conditions with DeepLoc annotated median normalized signal intensity data of membrane protein kinases clustered based on Euclidean distance. Greyed out areas represent missing values.

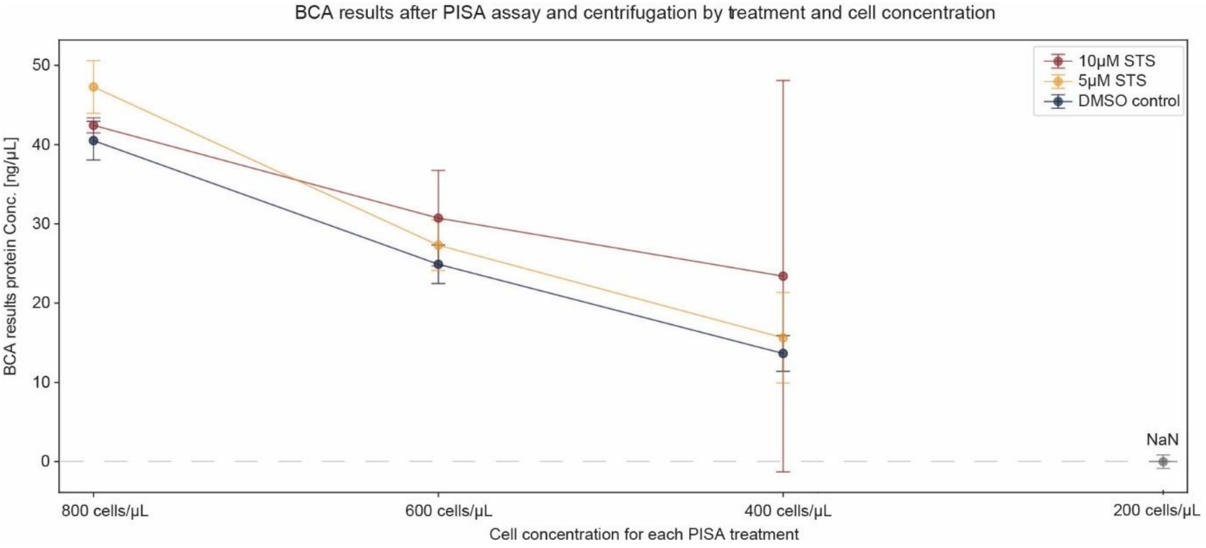

Supplementary Figure 2: BCA assay results showing protein concentration (ng/µL) across cell concentrations from 800 to 200 cells/µL, comparing 10 µM and 5 µM STS treatments as well as a DMSO control. Each point represents the average mean of four replicates (n=4), with error bars indicating ± standard deviation of the mean. Bar plots of the average number of identified proteins

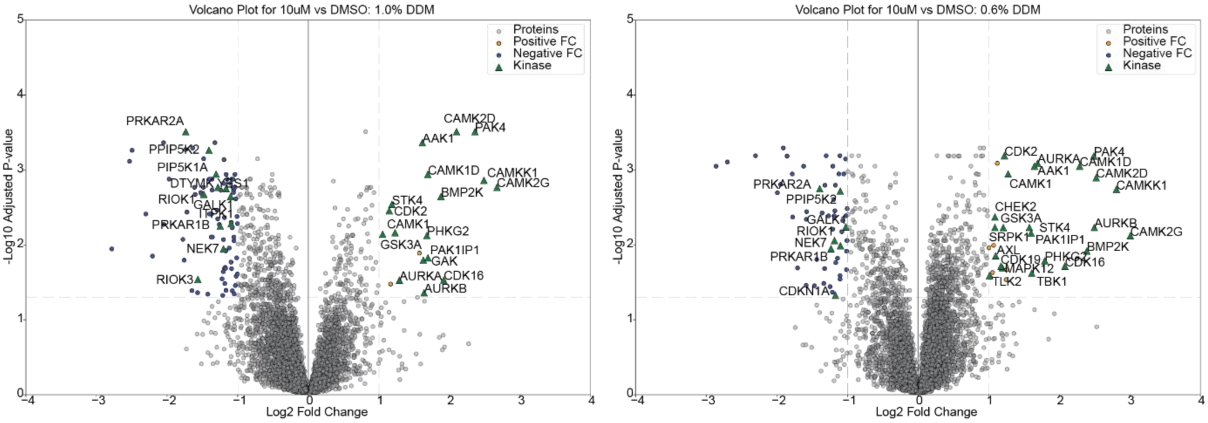

Supplementary Figure 3. Volcano plots of 1.0% and 0.6% DDM concentrations after pooling samples from PISA assay. Used on HeLa cells treated with 10 µM STS against DMSO. Depicted are the -log10 BH adjusted p-values of all proteins between treatment and control against the log2 FC of all proteins.

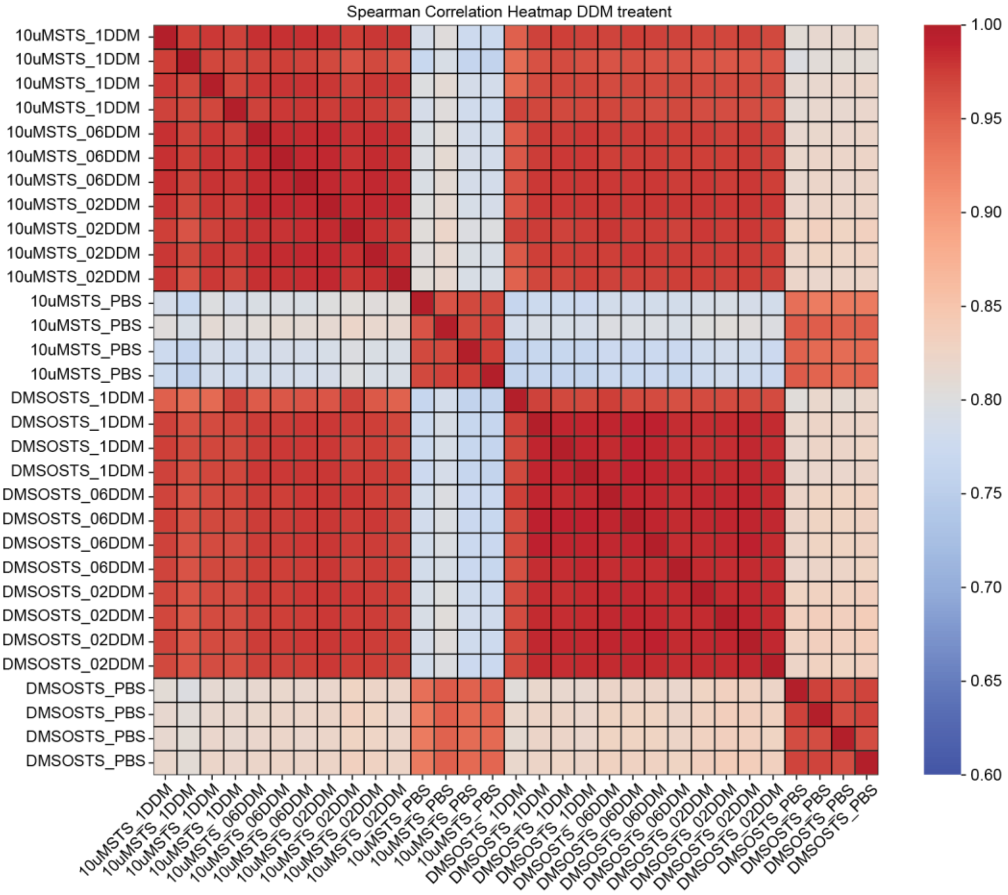

Supplementary Figure 4. Correlation heatmap between different DDM concentrations based on the median normalized and log2 transformed signal intensity for each replicate.

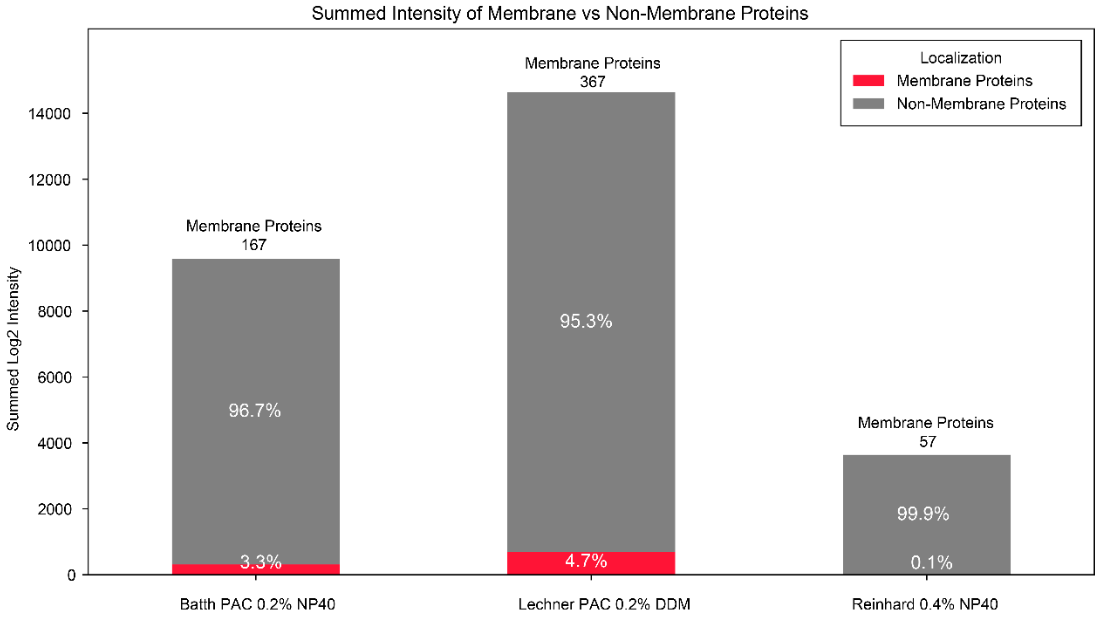

Supplementary Figure 5. Stacked plot of summed log2 intensities of membrane and non-membrane protein based on 3 different datasets of HeLa cells treated with DMSO from Reinhard et al. Batth et al. and Lechner et al.

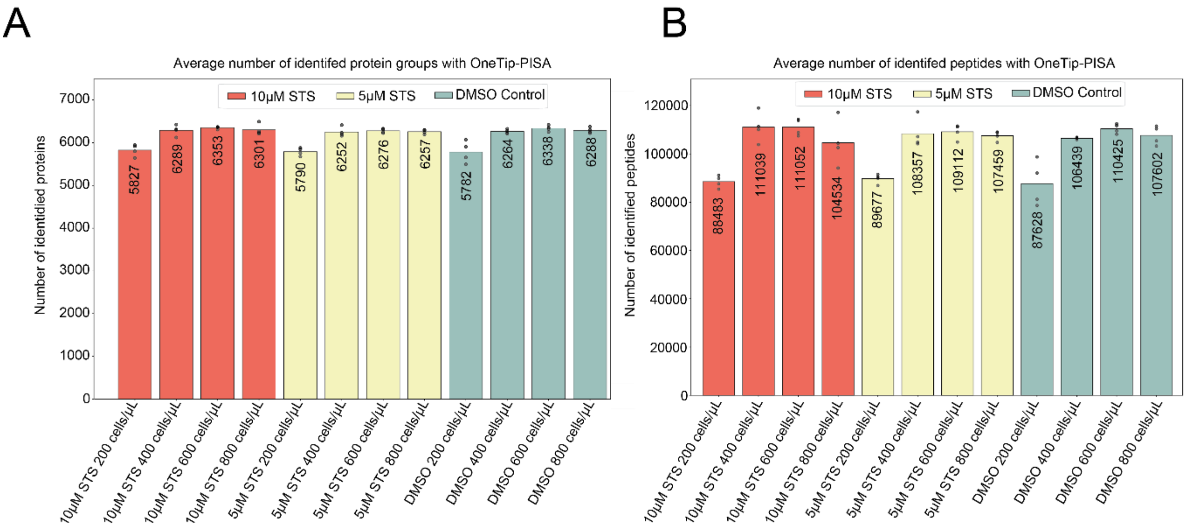

Supplementary Figure 6: (**A**) Proteins and peptides identified (**B**) for each STS treatment and control across cell dilutions from 200 to 800 cells/µL, with individual replicate values shown as dots (n=4).

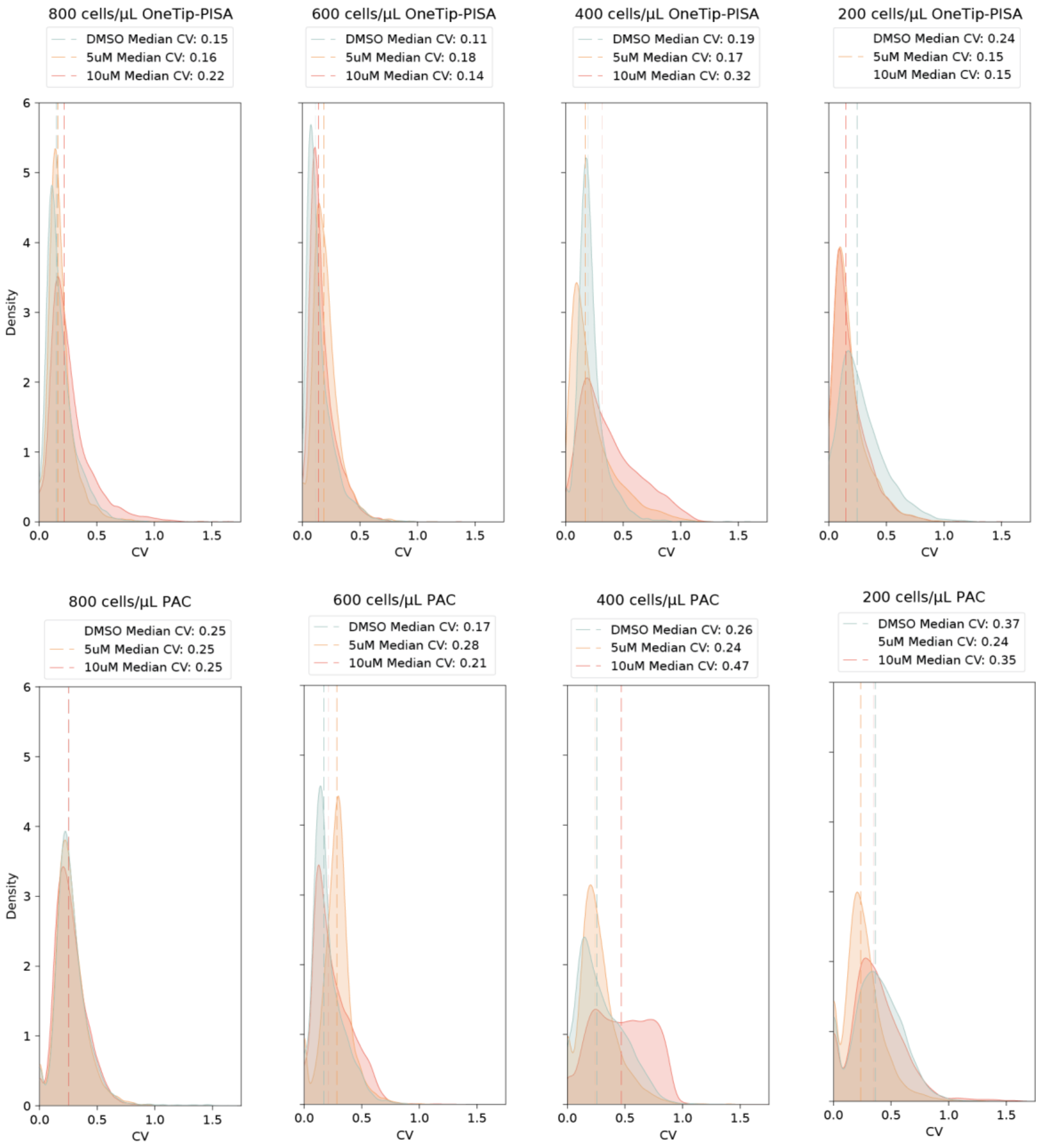

Supplementary Figure 7. CV values of all proteins between 800, 600, 400 and 200 cells/µL between PAC and One-Tip-PISA considering all proteins. Median CV value between all treatments and cell concentrations are depicted in each plot.

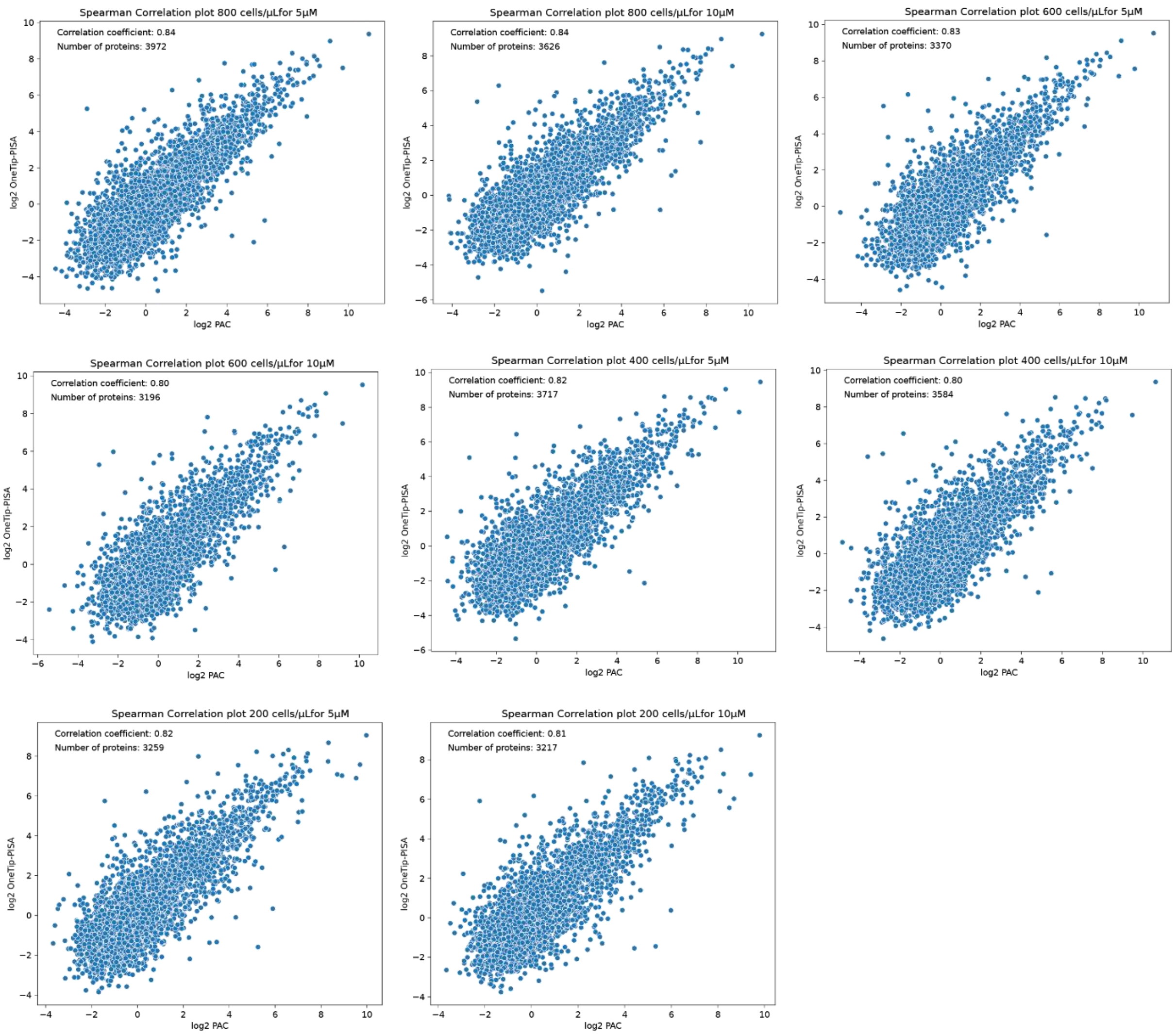

Supplementary Figure 8. Spearman correlation plots between One-Tip-PISA method against the PAC method. All proteins have been used as median normalized log2 transformed signal intensities with number of used proteins and spearman correlation depicted in the plot.

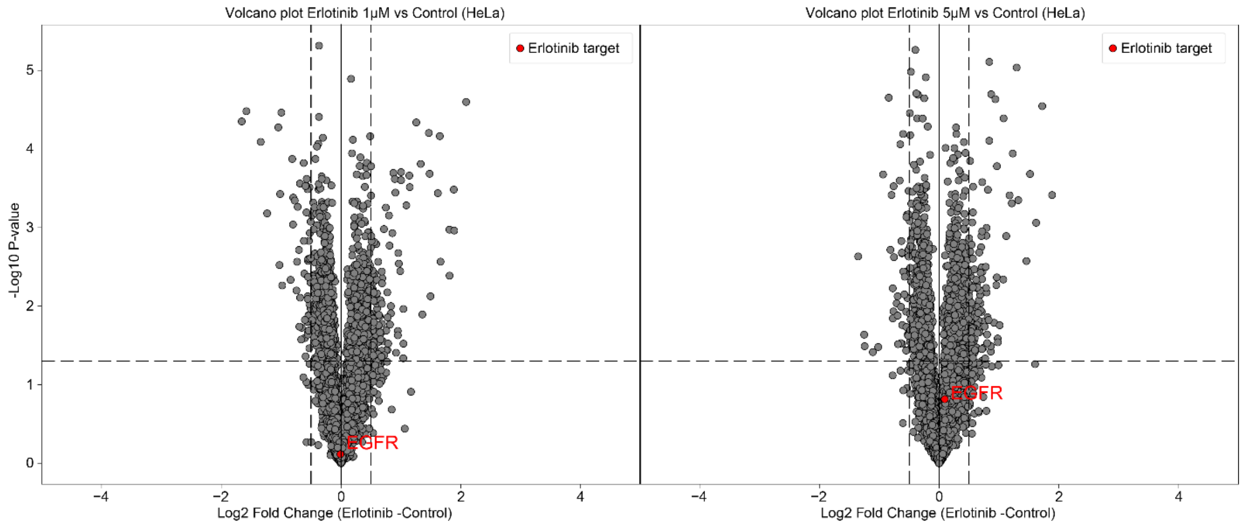

Supplementary Figure 9: Volcano plots of HeLa cells treated with 1 and 5 µM Erlotinib comparing log2FC between treatment and control against the -log10 p-value with highlighted kinase target.

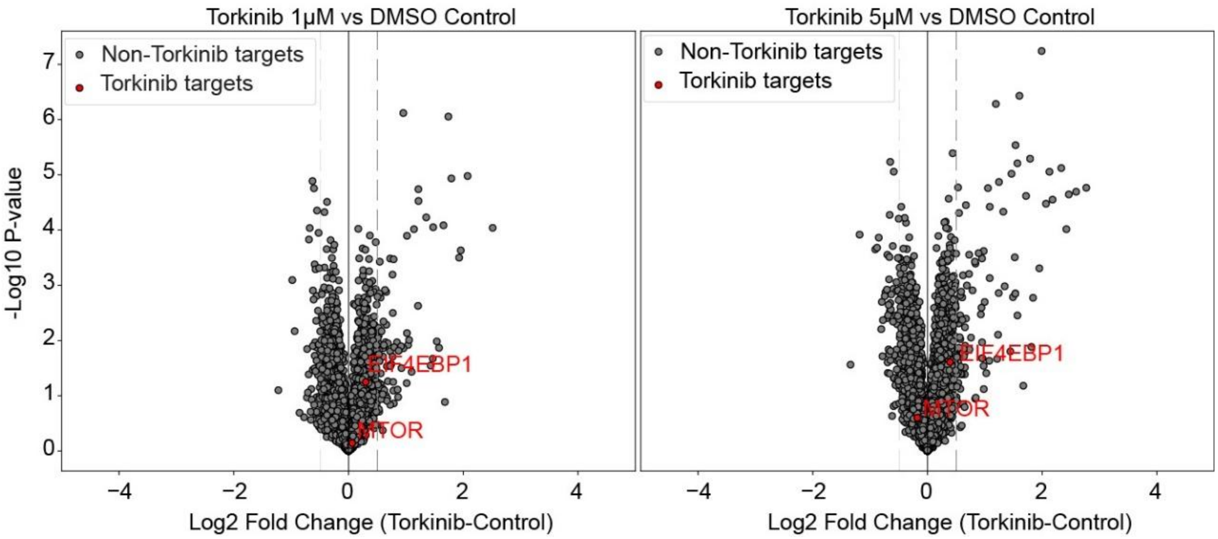

Supplementary Figure 10: Volcano plots of HeLa cells treated with 1 and 5 µM Torkinib comparing log2FC between treatment and control against the -log10 p-value with highlighted kinase targets.

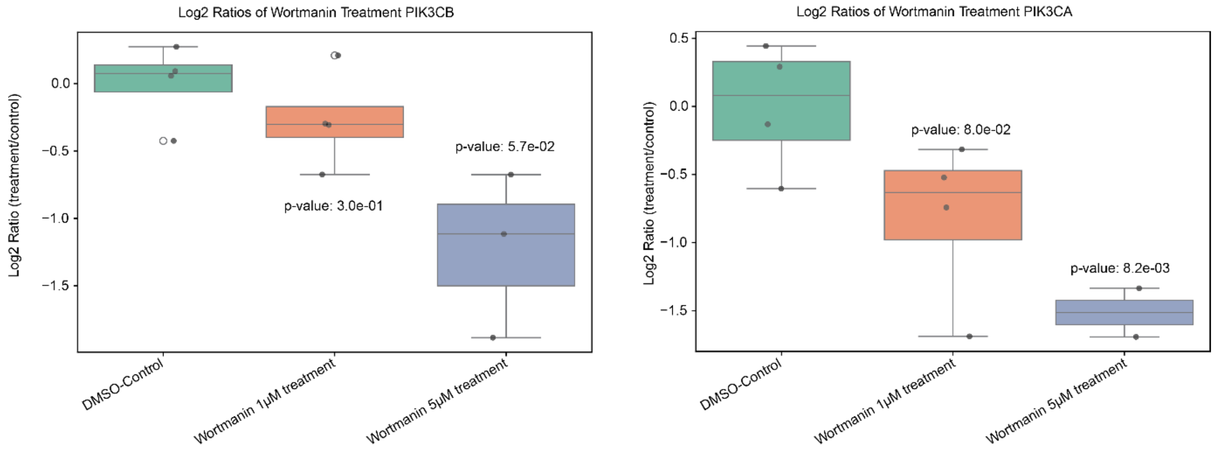

Supplementary Figure 11: Log2 Ratios between treatment and control of 1 and 5 µM Wortmanin treated HeLa cells and its known targets PIK3CB and PIK43CA. Welch t-test was performed between treatment and control and the resulting p-values have been added to each boxplot.

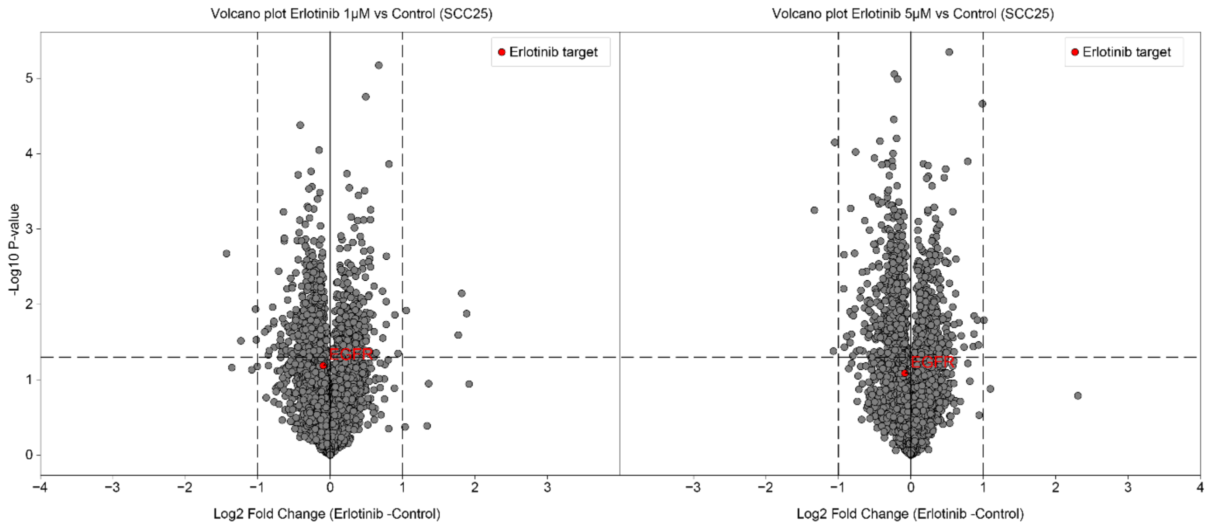

Supplementary Figure 12: Volcano plots of SCC25 cells treated with 1 and 5µM Erlotinib comparing log2FC between treatment and control against the -log10 p-value with highlighted kinase target.

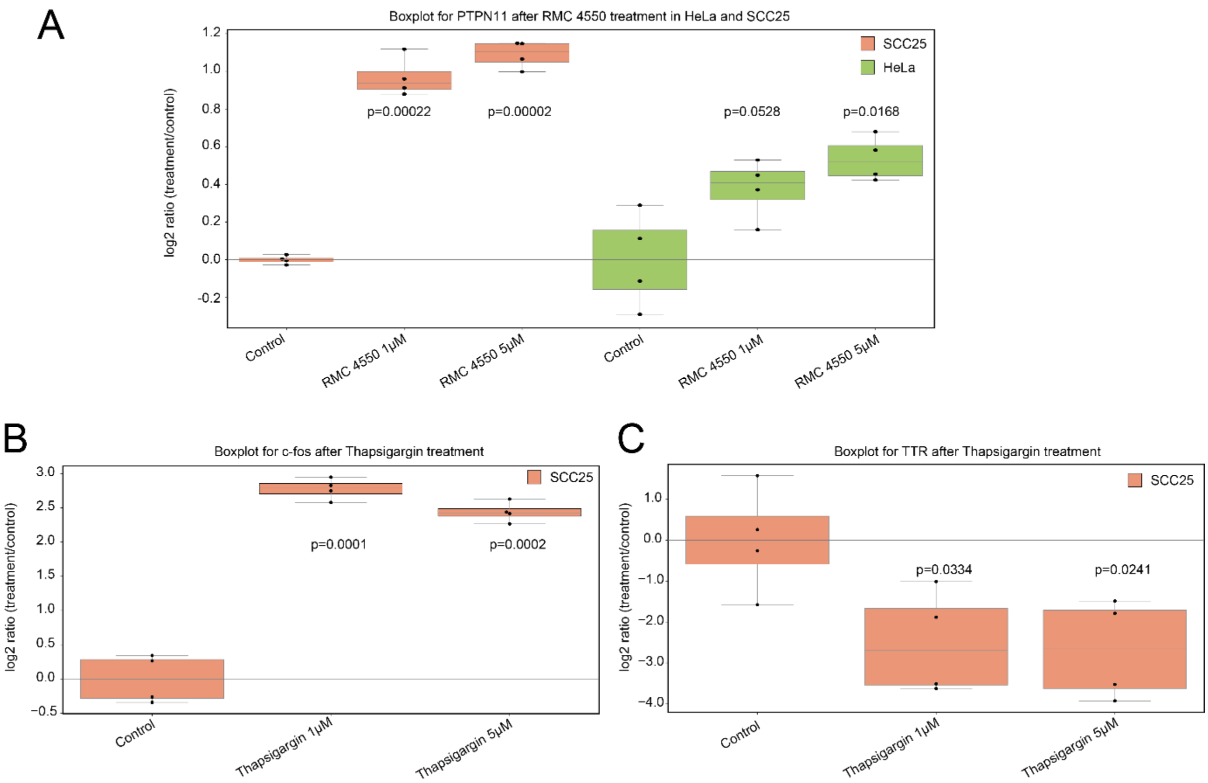

Supplementary Figure 13:(A) Boxplots of log2-transformed signal intensity ratios of the tyrosine phosphatase SHP-2 (PTPN11) for 1µM and 5 µM RMC-4550 treatment conditions. The log2 ratios are calculated as the difference between the log2-transformed and median normalized protein intensities under treatment conditions and their DMSO Control. Each treatment was performed in 4 replicates and are represented as black dots in the boxplots. Statistical significance between conditions and control was determined using Welch's t-test, with p-values displayed above each boxplot. (**B and C)** Boxplots of log2-transformed intensity ratios of the proteins c-fos and TTR for 1µM and 5µM Thapsigargin treatment conditions. The log2 ratios are calculated as the difference between treatment and control.

Supplementary Table 1. One-way ANOVA result table of specific protein localizations comparing DM lysis conditions

Location	F-value	P-value	SS Between	SS Within	DF Between	DF Within	MS Between	MS Within	Significant
Cell membrane	289	9.72E-11	145815	1851	3	11	48605	168	Yes
Cytoplasm| Cell membrane	146	3.91E-09	1943	49	3	11	648	4	Yes
Cell membrane |Lysosome/Vacuole	314	6.13E-11	16868	197	3	11	5623	18	Yes
Mitochondrion	599	1.83E-12	324558	1987	3	11	108186	181	Yes
Endoplasmic reticulum	1134	5.55E-14	336152	1086	3	11	112050	99	Yes

Supplementary Table 2. One-way ANOVA result table of specific protein localizations comparing PAC and OT-PISA method

Location	F-value	P-value	SS Between	SS Within	DF Between	DF Within	MS Between	MS Within	Significant
Cell membrane	12	0.000132	4227	2444	3	20	1409	122	Yes
Cytoplasm	67	1.24E-10	160291	15864	3	20	53430	793	Yes
Nucleus	3	0.0437	23644	48611	3	20	7881	2431	Yes
Mitochondrion	16	1.88E-05	14738	6327	3	20	4913	316	Yes
Endoplasmic reticulum	2	0.09	4570	12252	3	20	1523	613	No
Extracellular	3	0.0621	126	292	3	20	42	15	No
Cytoplasm|Nucleus	46	3.81E-09	70020	10189	3	20	23340	509	Yes

Supplementary Table 1 and 2. 1: One-way ANOVA table illustrating the significance of localization-based protein intensity differences, with metrics including sum of squares (SS), degrees of freedom (df), mean square (MS), F-values, and p-values ($p < 0.05$ for significance). **2:** One-way ANOVA table summarizing variance across cellular localizations, with significance determined by p-values < 0.05; critical F-values were calculated using between and within-group sum of squares, mean squares and degrees of freedom.
