# Supplementary material for: High-throughput proteome integral solubility alteration assay for low cell input using One-Tip
Source: Commun Chem. 2025 Sep 26;8:282. doi: 10.1038/s42004-025-01670-4 (PMC12474936; doi:10.1038/s42004-025-01670-4)
Supplement: Supplementary file 3 — Description of Additional Supplementary Files [file 42004_2025_1670_MOESM3_ESM.pdf]

Description of Additional Supplementary Files

File name: Supplementary Data 1

Description: Source data underlying the graphs and charts
